# The differential role of socioeconomic status dimensions in depressive symptoms of aging adults: data from the Hamburg City Health cohort Study

**DOI:** 10.3389/fpubh.2024.1430325

**Published:** 2024-08-29

**Authors:** Anne Klimesch, Leonie Ascone, Axel Schmager, Elina Petersen, Hanno Hoven, Olaf von dem Knesebeck, Jürgen Gallinat, Simone Kühn

**Affiliations:** ^1^University Medical Center Hamburg-Eppendorf, Center for Psychosocial Medicine, Department of Psychiatry and Psychotherapy, Hamburg, Germany; ^2^University Medical Center Hamburg-Eppendorf, Epidemiological Study Center, Hamburg, Germany; ^3^University Medical Center Hamburg-Eppendorf, Institute for Occupational and Maritime Medicine, Hamburg, Germany; ^4^University Medical Center Hamburg-Eppendorf, Center for Psychosocial Medicine, Institute for Medical Sociology, Hamburg, Germany; ^5^Center for Environmental Neuroscience, Max Planck Institute for Human Development, Berlin, Germany

**Keywords:** socioeconomic disparities in health, social class, depression, patient health questionnaire, mental health, cohort studies, health inequalities, health transition

## Abstract

**Background:**

Socioeconomic status (SES) has consistently been associated with depressive symptoms, however, it remains unclear which subset of SES variables is most relevant to the development of depressive symptoms. This study determined a standardized SES-Index to test the relationship of its sub-dimensions with depressive symptoms.

**Methods:**

HCHS data (*N* = 10,000; analysis sample *n* = 8,400), comprising participants 45+ years of age, was used. A standardized approach to quantify SES was employed. Depressive symptoms were quantified using the Patient Health Questionnaire-9 (PHQ-9). Using multiple linear regression models, PHQ-9-scores were modeled as a function of age and sex, and (1a) total SES-Index score versus (1b) its three sub-dimension scores (education, occupational status, income). Models were compared on explained variance and goodness of fit. We determined risk ratios (RR, concerning a PHQ-9 sum score ≥ 10) based on (low, middle, high; 2a) SES-Index scores and (2b) the sub-dimension scores, with groups further differentiated by sex and age (45–64 versus 65+). We distinguished between the total SES-Index score and its three sub-dimension scores to identify relevant SES sub-dimensions in explaining PHQ-9-variability or risk of depression.

**Results:**

Among all regression models (total explained variance 4–6%), income explained most variance, but performance of the SES-Index was comparable. Low versus high income groups showed the strongest differences in depressive trends in middle-aged females and males (RRs 3.57 and 4.91). In older age, this result was restricted to females (RR ≈ 2). Middle-aged males (versus females) showed stronger discrepancies in depressive trends pertaining to low versus high SES groups. In older age, the effect of SES was absent. Education was related to depressive trends only in middle-aged females and males. In an exploratory analysis, marital status and housing slightly increased model fit and explained variance while including somatic symptoms lead to substantial increases (R^2^_adj_ = 0.485).

**Conclusion:**

In line with previous research, the study provides evidence for SES playing a significant role in depressive symptoms in mid to old age, with income being robustly linked to depressive trends. Overall, the relationship between SES and depressive trends appears to be stronger in males than females and stronger in mid compared to old age.

## Background

Socio-economic status (SES) has been widely investigated in research on (mental) health-related inequality concerning three main variables: educational qualification, occupational status, and income levels—either at the individual or at the household level (1–4). In social epidemiological research, theoretical origins of these variables have been explained (4). For education, the common rationale is that it may capture the intergenerational transition of SES and reflects intellectual resources and potentials (5). Knowledge and skills reflected in educational qualification may in turn represent underlying cognitive capacities that relate to health literacy, health-related behavior, and service use (6). One’s occupation reflects social standing or prestige. Different types of occupation harbor both potential stressors and opportunities. For instance, occupation-related privileges include access to social circles and connections that allow for better access to housing, healthcare, or personal opportunities. On the other hand, one’s occupation may pose significant health risks, such as exposure to toxins, high social stress, working under time pressure, or hard physical labor (7, 8). Finally, income can influence a wide variety of circumstances that are related to (mental) health (e.g., housing, being able to afford going on vacation, having a healthy diet and lifestyle, or access to healthcare) (9).

Depression is on the rise worldwide. In 2017, depression affected 4.4% of the global population, making depressive disorders the leading cause of disability (10). The COVID-19 pandemic further enhanced the prevalence of depressive disorders, with an estimated increase of 27.6% pre-to-mid pandemic (11). In Germany, a population-based prevalence in adults of 10.1% (women: 11.6%, men: 8.6%) has been reported (12), and increasing age has been associated with a less favorable course of a major depressive disorder within 2 years by a Dutch cohort study (13). Thus, to further study depressive symptoms with adequate samples and methods at different sites and thereby attempt to identify relevant individual characteristics from the background of social disparities is an important endeavor to inform (local) policy and healthcare.

The association between SES and depression in adults is well-studied and generally robust. A Spanish cohort study involving older adults showed that the risk experiencing at least two depressive symptoms was significantly higher in those with incomplete primary education compared to those with completed secondary education (χ^2^ = 40.25; *p* < 0.001) as well as those experiencing financial hardship compared to those with subjective financial security (X^2^ = 23.62; *p* < 0.001) (14). A German national health survey conducted with participants between 18 and 79 years of age (*N* = 7,485) showed that SES-related discrepancies in depression rates were largest in middle-aged participants, especially women. In age group 30–44 years, depression prevalence was higher for individuals with low SES (females: 21.2%, males: 15.7%) compared to medium (9.9, 4.2%), and high SES (6.0, 3.6%). In age groups 45–64 and 65–79 years, the discrepancies were slightly lower, but still marked (15). International studies confirm differential depression prevalence by SES. For instance, the Iranian Neyshabur longitudinal study that investigated adults aged 50–94 years has shown that, among other factors, low education was a relevant predictor of depression, with depression prevalence in a low SES group (12.2%) being significantly higher than in a high SES group (6.5%) (16). In a 13-year follow-up of the French GAZEL cohort study, individuals between 59 and 69 years of age with low or intermediate occupational status were more likely to exhibit depression and worse trajectories concerning symptomatic progression compared to individuals with a high occupational status. A British meta-analysis showed that treatment trajectories of a sample of *N* = 4,864 patients with a mean age of 42.45 (SD = 14.0) were affected by socioeconomic variables. At 3–4-month assessments, independent of treatment, depression scores were 28% (95% CI, 20–36%) higher in unemployed individuals compared to employed individuals and 18% (95% CI, 6-30%) higher in individuals with alternative housing status (e.g., non-ownership housing, living with family members or friends, living in hostels, being homeless) compared to individuals owning a home. Similar patterns emerged at 6-8-month assessments (17). Women are more likely to experience depression than men (1, 18, 19). For example, in the GAZEL study, observed rates were 41.9% in women compared to 28% in men. Older age (20, 21) and female gender (22) are generally and inter-culturally related to lower SES.

**Figure 1:**
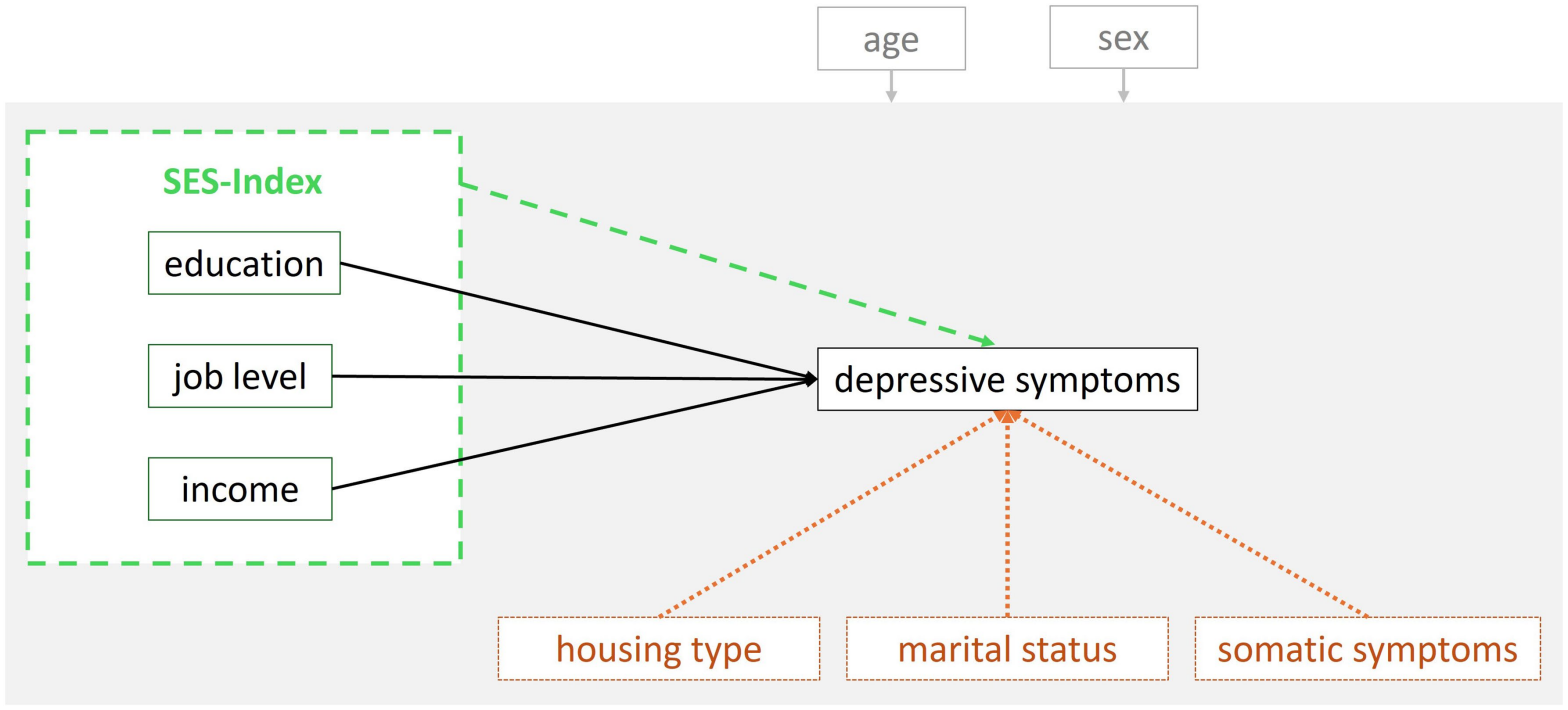
Hypothesized relationships that were tested and compared in the present study. Research question (1): Comparison of the SES-Index variable (dashed green line) with its sub-dimensions (solid black lines). Exploratory research questions (3a, b): Comparison/ robustness check of the variables in research question (1) with three additional SES variables (dotted orange lines). The hypothesized effect of age and sex (covariates) on every variable in the model is represented in gray.

Throughout the literature on depression, definitions and operationalizations of SES vary, often lacking a clear rationale for selecting a specific set of SES variables. This may be due to country-specific particularities concerning SES of the examined population. In Germany, an SES-Index assessment and computation approach has been developed and established in large cohort studies, relying on the above-mentioned three main SES sub-dimensions: *education*, *occupational status* (hereafter referred to as: *job status*) and household net equivalent *income* (23). The present study aimed at evaluating the performance of this standardized SES-Index approach by examination of the association between SES and current depressive symptoms in data of the Hamburg City Health Study (HCHS; *N* = 10,000). The aim of HCHS is to identify prospective somatic and psychological risk and resilience factors for common physical and mental disorders in the aging population (24). Participants of the HCHS are between 45 and 74 years of age and randomly drawn based on public registers from the general population of the city of Hamburg, Germany. In the present study, there were two main research questions (Figure 1):

1. In a linear regression approach, how much of the variance in depressive symptoms can be explained by: (1a) a global SES-Index measure compared to (1b) specific SES sub-dimensions (education, job status, and income), while controlling for age and sex?
2. To what extent are different levels (low, medium, high) of both the global SES-Index and its sub-dimensions (education, job status, income) associated with differential risk ratios of clinically relevant levels of depressive symptoms in separate age- and sex-stratified groups?

As exploratory analyses (which are documented in the Supplementary material, SM), the present study set out to (3a) assess whether there were additional SES variables relevant in explaining variance in depressive symptoms.

Moreover, (3b) the role of somatic symptoms was assessed to put the variance explained by the SES sub-dimensions and other SES variables into perspective (i.e., *comparative relevance* of SES compared to a strongly associated risk-factor for depression) and to check for the *robustness* of SES in explaining variance in depressive symptoms (Figure 1). Poor self-rated physical health has been identified to be among the strongest associated risk factors in a large machine-learning-based analysis (*N* = 67,603) in middle-aged and older adults which concurrently tested a broad range of depression-related social, health, functional, and cognitive risk/ protective factors (25).

## Methods

The present study used first wave cross-sectional data (*n* = 10,000) which were randomly sampled using inhabitant records stratified by age and sex and collected between 2016 and 2022. Participants underwent a 7-h interdisciplinary examination involving, among others, the standardized acquisition of demographic and SES data as well as psychological questionnaires. The study design and data acquisition process of the HCHS, which aims at a total sample size of *n* = 45,000, have been described in detail by Jagodzinski et al. (24).

### Measures

#### Socio-economic status index

To assess the socioeconomic status, we computed an SES-Index as previously implemented in the “*Measurement of the socioeconomic status within the German Health - Update 2009*” [GEDA; (26)], and “*Measurement of socioeconomic status in the German Health Interview and Examination Survey for Adults*” [DEGS1; (23)]. These two large representative German population studies are part of the national health monitoring and have been performed by the Robert Koch Institute in adults (>18 years). The sum of the three index sub-scores, all ranging from 1.00 to 7.00, forms the SES-Index total score: *education* (school- and professional education), *job status* (type and characteristics of one’s occupation) and *income* (net equivalent household income), (27). The advantage of the index is that it uses the same scale for all sub-scores, facilitating comparability. In addition, the sub-scores can be considered metric, as they allow for deviations to the first decimal, which facilitates analyses of status groups and application of each sub-score in distinct analyses. The factorial validity and reliability of the index was successfully replicated within the scope of the present study (details can be found in the SM). To enable replicability of the SES-Index for future studies, code for the development of the index is shared in a repository.^1^ In the following, the variable assessment within HCHS and formation of the sub-scores is described in detail for each sub-dimension: *education*, *job status*, and *income*.

#### Socio-economic status index—subscale education

Education was assessed following the procedure of the GEDA (26) which applied the ‘*Comparative Analyses of Social Mobility in Industrial Nations’* (28) (CASMIN) scheme. The CASMIN classifies nine educational classes based on combinations of an individual’s highest obtained school degree plus professional education level (e.g., vocational training certificates, diploma, Master’s degree). For each of the different educational classes, a specific index value is assigned. Supplementary Table 1.1 displays how the data on education were classified and coded, and Supplementary Table 1.2 displays how the codes were subsequently scaled to form the SES-Index sub-score *education*. In case individuals indicated multiple degrees, the highest one was considered.

#### Socio-economic status index—subscale job status

Participants were asked to indicate which category (e.g., unlearned laborer, freelancer/ company owner, senior official) their current - or, if currently unemployed or retired, last occupation - belonged to. Depending on the indicated category, further specifications on the occupational level or rank were assessed (e.g., responsibilities for resources or personnel, hierarchical position). Supplementary Table 1.3 provides an overview of the occupational category and level or rank combinations and the assigned SES-Index points which are based on the revision of the ‘*International Socio-Economic-Index of Occupational Status*’ (29) conducted in the GEDA study (26).

#### Socio-economic status index—subscale income

Participants were asked to indicate their monthly household net income using 17 categories (<500€, 500€ to <750€, 750€ to <1,000€, 1,000€ to <1,250€, 1,250€ to <1,500€, 1,500€ to <1,750€, 1,750€ to <2,000€, 2,000€ to <2,250€, 2,250€ to <2,500€, 2,500€ to <3,000€, 3,000€ to <3,500€, 3,500€ to <4,000€, 4,000€ to <4,500€, 4,500€ to <5,000€, 5,000€ to <6,000€, 6,000€ to <8,000€, ≥8,000€). In addition, participants were asked to indicate (a) the total household size (number of income dependent and/or contributing individuals including oneself), and (b) the number of adults (age > 14 years) living in the household including oneself. The number of children thus equals the difference between the responses to (a) and (b).

The class mean of each of the 17 net household income categories was determined. Then, it was transformed into the *OECD-modified scale* corrected net equivalent household income (30). In this procedure, the respondent receives a weight of 1.0, each additional adult (>14 years) receives a weight of 0.5 and each child (<14 years) receives a weight of 0.3. The household income is divided by the sum of the person weights. The resulting *OECD-modified scale* approximates the truly disposable income of the respective participant being a member of a larger household, which makes single and multiple-person-households better comparable. The empirical data distribution of the *OECD-modified scale* was (roughly) evenly split into 13 categories and these were assigned an SES-Index value in ascending order in steps of 0.5 (26). The resulting 13 categories (incl. Respective net equivalent household income spans) can be found in Supplementary Table 1.4.

#### Individual income

Individual net income was assessed using the same 17 categories that were applied for household net income. No further data transformation was conducted. Individual income was considered as an alternative to the net equivalent household income. Since individual income is more directly linked to the queried person, it was deemed relevant to compare the computed net equivalent household income index value with this more proximal income value in explaining variance in depressive symptoms.

#### Depression

The PHQ-9 is a screening tool that comprises nine items assessing the DSM-IV criteria for a depressive episode on a four-point Likert scale each (0 = not at all, 1 = several days, 2 = more than half of the days, 3 = nearly every day) and demonstrated high internal consistency (α = 0.86–0.89) and strong test–retest variability (*r* = 0.84) (31). The German version of the PHQ-9 has been validated in a general population sample demonstrating high convergent validity with the General Health Questionnaire-12 (*r* = 0.59, *p* < 0.0001) and with the Beck Depression Inventory (*r* = 0.73, *p* < 0.001) (32). For the linear data analysis approach, we used the sum scores to capture the full range of depressive symptom severity present in the sample. For the frequency rate estimation by low, medium, and high SES groups (risk ratio approach) the sample was classified into ‘no depression’ (sum score ≤ 9) versus ‘(potential) presence of a major depressive disorder’ (sum score ≥ 10) with the aim to validate results from the continuous approach and simplify interpretation regarding risk factors of a major depressive disorder. The cut-off of 10 (moderate depression level) is common practice for the PHQ-9 and its diagnostic properties have been confirmed in a meta-analysis (33). For reasons of efficiency, PHQ-9 scores above nine are referred to as ‘depression’ hereafter.

### Statistical analyses

All analyses were run in IBM SPSS Statistics for Windows [Version 28.0.1.1 (34)]. A series of multiple linear regression models were carried out, with all analyses being controlled for age and sex, to estimate the additional (1a) overall explained variance in depressive symptoms by a generic SES-Index, and (1b) identify which SES sub-scores (*education, job status, income*) would drive the effects and hence best explain variance in depressive symptoms. In a ‘baseline model’ (model 1), depression was modeled as a function of age and sex (first regression predictor block). In model 2, the total SES-Index was added as predictor to explore its performance in explaining variance in depressive symptoms over and above age and sex. In model 3–5, the three sub-scores were added separately in the second regression predictor block, to explore the performance of each SES sub-dimension individually in explaining variance in depressive symptoms. Finally, to determine the competitive performance of all three sub-scores against one another, they were simultaneously added in the second predictor block in model 6. Additionally, to compare the performance of household net equivalence *income* that is part of the SES-Index versus individual income in explaining variance in depressive symptoms, individual income was added as a predictor variable in model 7. All models used the exact same sample to assure comparability of results (*n* = 8,400; also see ‘Missing data and multiple imputation’ section for details).

To avoid variance inflation of regression coefficients and to test the stability of estimates of regression, the following parameters were considered: coefficients, sample size, proportion of explained variance, variance of the predictors, and variance inflation factor (VIF) values (35). Multicollinearity of each included predictor with the other predictors was carefully evaluated. The performance of the different models was compared using the Bayesian Information Criterion (BIC), which favors parsimonious models over more complex ones, whereby lower values indicate a better fit. The rationale to use the BIC was to derive the most powerful SES regressor variables explaining variations in depressive symptoms by directly comparing BIC values of the respective models.

To (2) validate the usefulness of the SES-index, it was tested whether having a low, medium, or high SES was associated with different depression rates across ‘social strata’. Chi-square tests comparing depression rates (dichotomous variable using a clinically validated cut-off) across sex and age groups (i.e., 45–64 vs. ≥ 65 years), as well as the three SES-levels (low, medium, high), were conducted both for the global SES-Index and for the sub-scores (*education*, *job status*, *income*).

As (3) additional exploratory analyses, we (3a) entered additional available SES variables (housing type, marital status) in addition to the control variables age and sex and the three sub-scores (education, job status, income) to the model to check whether there were potentially more SES variables relevant in explaining variance in depressive symptoms and how much variance they would explain beyond the three ‘standard’ SES sub-dimensions. In addition, (3b) to put the amount of explained variance by the SES sub-dimensions and other SES variables into perspective (i.e., *comparative relevance* of SES compared to a strongly associated risk-factor for depression) as well as to check whether SES remained to be significant (*robustness* of SES in explaining variance in depressive symptoms), somatic symptoms [sum score, using the German version of the PHQ-15 (36)] were added in a final step. To assure comparability, these additional exploratory linear regression models (method: enter; model 1 predictors: age, sex, education, job status, income; model 2: all predictors from model 1 + housing and marital status; model 3: all predictors from model 2 + somatic symptoms) were computed within a sub-sample with complete data on all additional variables (*N* = 6,341) to the already (previously imputed) SES-Index subscore data.

### Missing data and multiple imputation

Multiple imputation was restricted to those cases that had data for at least one SES-Index sub-score (which was the case for 92.8% of all cases, i.e., *n* = 718 cases had no sub-score data at all). In addition to the sub-scores, age, and sex (both had complete data) were used as predictors to impute missing SES sub-score data. The SES-Index sub-score *income* and the variable individual income had a high percentage of missing data (33.9 and 24.1%, respectively of the *N* = 10,000 initial sample), which is, however, a common problem in the acquisition of data on income and wealth reported in the literature (37). Concerning *education*, 14.1% of the data was missing, and for *job status* 23.7% of the data was missing for different reasons (for missing data patterns of the SES-Index sub-scores, see Supplementary Tables 2.1, 2.2). Multiple imputation was then conducted with ten imputations per participant using the automatic; automatic method selection implemented in SPSS (38), which chooses the imputation method based on the characteristics of the data. The 10 imputed values were then averaged to generate a single value per person. Data on depressive symptoms were not imputed. Data on individual income was imputed in a separate step, including age, sex and the SES-Index sub-scores (unimputed data). The reason for this differentiated imputation approach was that a primary goal of the study was to provide a standardized SES dataset with an SES-Index and imputation approach independent of other research questions for adoption across HCHS research. The analyses on depressive symptoms in the sample were done in a next step to test the performance of the SES-Index and sub-scores, as well as individual income. Only 11% of data were missing for the PHQ-9, thus, the dependent variable was not imputed. A constant sample size was crucial for being able to compare the different regression models on individual vs. net equivalent household *income*, which further informed the decision on the above-described imputation approach.

## Results

### Sample characteristics

**Figure 2:**
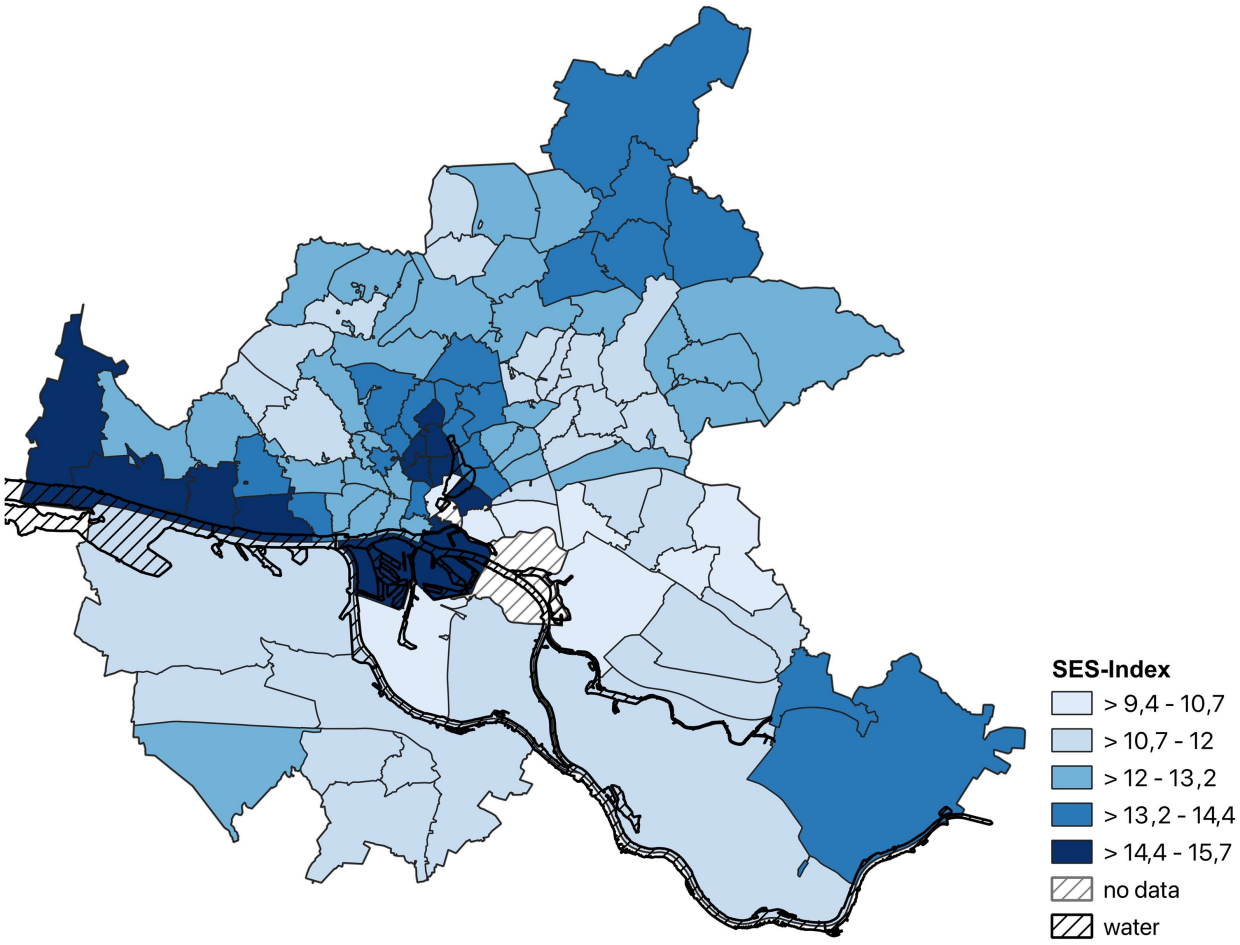
Map of the city of Hamburg (Germany) displaying the distribution of the SES-Index in the final analysis sample (*n* = 8,400) across districts.

Figure 2 displays the SES-Index distribution across the districts of the city of Hamburg (possible SES-Index range: 3–21). In the sample of analysis (*n* = 8,400), there was a near equal distribution of male and female sex: n_male_ = 4,138; n_female_ = 4,262. Individuals were 62 years old on average (SD = 8.40; age range: 46–78). Of the *n* = 8,213 available data on household size, *n* = 1,986 (≈24.2%) were single households, and *n* = 6,227 (≈75.8%) were multiple person households. Of the multiple person households, *n* = 855 (≈13.7%) reported children living in the household who were 14 years or younger.

Examining the raw data revealed that 34.1% (3,406/ 8,400 cases) had the highest school degree within the German education system (*‘Abitur’*, comparable to *A-levels* in Anglo-Saxon countries), which resembles the percentage in the total German population in 2019 (> 15 years; 33.5%) (39). The sub-score mean for *education* was M = 4.67 (SD = 1.56; scale range: 1–7). *Job status* was moderate to high on average in the sample (M = 4.0, SD = 1.16; scale range: 1–7). The sample mean on the sub-score SES-Index *income* (M = 4.06, SD = 1.66; scale range: 1–7) and the sample mean of individual net income (M = 7.78, SD = 3.67; scale range: 1–17) represent values between 1,750€-2,000€ and 2,000€-2,250€, respectively. The mean individual net income of the sample is thus roughly comparable to the mean individual net income of employed Germans in 2016–2018 (1,905–2,008€) (40). To assess the distribution of low, medium, and high SES-Indices, the sample was split into quintiles based on the total SES-Index (1^st^quintile = low, 2^nd^–4^th^quintile = medium, 5^th^quintile = high). Similarly, the sample was split into quintiles for each of the SES-Index sub-scores to classify ‘low’, ‘medium’, and ‘high’ status groups for each SES sub-dimension separately. The respective cut-off values are reported in Supplementary Table 2.3.

The PHQ-9 sample mean and standard deviation (M = 3.48, SD = 3.46) are comparable to the ones reported for the general German population in the literature (M = 3.30, SD = 3.65) (41). The dichotomized PHQ-9 variable “depression” (cut-off ≥10 on the PHQ-9) classified 530 (6.3%) individuals as depressed and 7,870 (93.7%) accordingly as not depressed. For further details concerning depression rates per sex and age groups, see section *Depression frequency rates by age, sex, and socioeconomic status*.

### Linear regression analyses

Checking the assumptions for linear regression revealed no meaningful deviations or violations. However, the plots indicated heteroscedasticity, i.e., slightly higher variability in residuals for higher levels of depression, which could narrow generalizability of the results to the population. Therefore, heteroscedasticity-consistent standard error estimators were applied in each regression. Before running the regression models, intercorrelations between all variables of interest were computed. The results can be found in Table 1. All independent variables (except sex) showed a weak negative association with the dependent variable. The negative association between the sub-score *education* and PHQ-9 sum score was very small but statistically significant. To look at this relationship more in detail, *education* was kept in the linear regression analyses despite the small correlation coefficient.

**Table 1:** Spearman rho intercorrelations between all variables of interest (*n* = 8,400).

	1	2	3	4	5	6	7
1. PHQ 9 sum							
2. Age	−0.134						
3. Sex^1^	0.186	−0.056					
4. SES-Index	−0.114	−0.115	−0.164				
5. Education	−0.040	−0.180	−0.094	0.813			
6. Job Status	−0.115	0.051	−0.168	0.743	0.524		
7. Income	−0.124	−0.093	−0.159	0.818	0.431	0.478	
8. Individual Income	−0.149	−0.136	−0.397	0.641	0.415	0.506	0.633

All correlation coefficients were highly significant with *p* < 0.001. ^1^0 = male, 1 = female.

**(1a): Performance of the SES-Index in explaining variance in depressive symptoms.** As can be seen in Table 2, the model including the SES-Index performed significantly better in explaining variance in depressive symptoms (model 2, 5.5% explained variance; BIC = 20,415) than age and sex alone (model 1 = baseline model, 4% explained variance; BIC = 20,532). This was also reflected in better goodness of fit of model 2 (∆BIC_2-1_ = −117), albeit being the more complex model.

**Table 2:** Model summaries of multiple linear regression analyses on depression (*N* = 8,400).

Model summary	*B*	*95%CI[B]*	*Robust S.E.*	*β*	*t*	*p*	R^2^_adj_	*BIC*
**1** [*F*(2, 8,397) = 178.06, *p* < 0.001]							0.040	20,532
Constant	6.01	[5.46 6.56]	0.274		21.41	<0.001		
Age	−0.050	[−0.058 −0.041]	0.004	−0.120	−11.23	<0.001		
Sex^1^	1.08	[0.930 1.22]	0.073	0.155	14.51	<0.001		
**2** [*F*(3, 8,396) = 162.81, *p* < 0.001]							0.055	20,415
Constant	7.94	[7.30 8.58]	0.343		24.28	<0.001		
Age	−0.055	[−0.063 −0.046]	0.004	−0.132	−12.40	<0.001		
Sex	0.932	[0.786 1.08]	0.075	0.135	12.49	<0.001		
SES-Index	−0.122	[−0.142 −0.100]	0.011	−0.122	−11.27	<0.001		
**3** [F(3, 8,396) = 131.24, *p* < 0.001]							0.044	20,505
Constant	6.99	[6.35 7.62]	0.331		21.57	<0.001		
Age	−0.054	[−0.063 −0.045]	0.004	−0.131	−12.08	<0.001		
Sex	1.02	[0.877 1.17]	0.074	0.147	13.70	<0.001		
Education	−0.144	[−0.191–0.097]	0.024	−0.065	−6.01	<0.001		
**4** [F(3, 8,396) = 141.85, *p* < 0.001]							0.048	20,475
Constant	7.02	[6.42 7.62]	0.310		22.96	<0.001		
Age	−0.048	[−0.056 −0.039]	0.004	−0.115	−10.79	<0.001		
Sex	0.999	[0.853 1.15]	0.074	0.144	13.43	<0.001		
Job status	−0.280	[−0.348 −0.213]	0.034	−0.088	−8.16	<0.001		
**5** [F(3, 8,396) = 173.25, *p* < 0.001]							0.058	20,385
Constant	7.57	[6.97 8.16]	0.320		24.94	<0.001		
Age	−0.055	[−0.063 −0.046]	0.004	−0.133	−12.28	<0.001		
Sex	0.925	[0.779 1.07]	0.075	0.134	12.44	<0.001		
Income	−0.282	[−0.326 −0.238]	0.024	−0.135	−12.53	<0.001		
**6** [*F*(5, 8,394) = 105.86, *p* < 0.001]							0.059	20,394
Constant	7.75	[7.11 8.40]	0.343		23.60	<0.001		
Age	−0.053	[−0.062 −0.044]	0.004	−0.127	−11.71	<0.001		
Sex	0.913	[0.767 1.06]	0.075	0.132	12.25	<0.001		
Education	0.013	[−0.045 0.072]	0.031	0.010	0.452	0.445		
Job status	−0.123	[−0.208 −0.038]	0.042	−0.044	−2.84	0.003		
Income	−0.252	[−0.302 −0.201]	0.027	−0.119	−9.80	<0.001		
**7** [F(3, 8,396) = 165.40, *p* < 0.001]							0.055	20,408
Constant	7.74	[7.12 8.36]	0.368		24.50	<0.001		
Age	−0.059	[−0.067 −0.050]	0.005	−0.142	−13.16	<0.001		
Sex	0.698	[0.540 0.856]	0.094	0.101	8.68	<0.001		
Individual income	−0.125	[−0.146 −0.104]	0.012	−0.136	−11.59	<0.001		

The independent variable of all models was PHQ-9 score. All models were fitted based on the same sample (*n* = 8,400). The t-statistics, *p*-values, and CIs were estimated based on robust standard errors. BIC, Bayesian Information Criterion. ^1^0 = male, 1 = female.

**(1b) (Competitive) performance of the SES-Index sub-scores (education, job status, income) in explaining variance in depressive symptoms.** It was found that model 6 including (sex + age +) all sub-scores (*education*, *job status*, *income*) at once (BIC = 20,394; 5.9% explained variance) and model 5 including (sex + age +) the *income* sub-score alone (BIC = 20,385; 5.8% explained variance) had similar goodness of fit indices and total amounts of explained variance (∆BIC_6-5_ = −9). In model 6, education was not significant and dropping it from the regression lead to a slightly improved BIC (20386) but an unchanged amount of explained variance (5.9%). Both models 6 and 5 were similar, but slightly superior over the (sex + age +) SES-Index score model 2, in terms of BIC and explained variance (∆BIC_6-2_ = −21, ∆*R*^2^_6-2_ = 0.004; ∆BIC_5-2_ = −30, ∆*R*^2^_5-2_ = 0.003), which was not the case for any of the other models with specific sub-scores instead of the generic SES-Index.

**(1c) Individual vs. SES-index income sub-score (based on net-equivalent household income) in explaining variance in depressive symptoms.** Individual income (model 7) performed less favorable than the *income* sub-score of the SES-Index (model 5; ∆_5–7_ = −23; ∆*R*^2^_5-7_ = 0.003).

### Depression frequency rates by age, sex, and socioeconomic status

Frequency rates in depression, defined based on a cut-off on the PHQ-9 data (≥10; total ‘depressed cases’ *n* = 530), significantly differed between age groups, females, and males. Frequency rates within middle age (45–64 years, *n* = 4,861) vs. older age (65+, *n* = 3,539) differed significantly with 7.9% vs. 4.1% [*X*^2^(1, 8,400) = 51.9, *p* < 0.001]. Thus, middle age was related to an about twice as high risk of depression, relative to older age (RR = 1.92). Of the total ‘depression cases’, *n* = 357 were female (8.4% of females [total *n* = 4,262]), and *n* = 173 were male (4.2% of males [total *n* = 4,138]). This difference was statistically significant (*X*^2^(1, 8,400) = 62.5, *p* < 0.001). Thus, female sex in the total sample was associated with a twice as high risk (RR = 2.0) for depression, relative to male sex.

In middle-aged females, low, medium, and high expressions of the total SES-Index, education, and income were related to significant differences in depression frequency rates (Table 3), in the sense of a ‘social gradient’. Job status only showed differences in frequency rates of depression between low and high expression groups. Concerning RRs, income and the total SES-Index descriptively differentiated most strongly between groups, with more than threefold relative risks for depression in the low vs. high SES groups (RR > 3.0). In older females, none of the SES variables except income was significantly related to differential depression frequency rates across SES groups, whereby low-income in females was related to a twice as high relative risk (RR = 2.15).

**Table 3:** Depression frequency rates (%) by age and socio-economic status categories in females (*n* = 4,262).

Total SES-Index					
	*Low total SES*	*Medium SES*	*High SES*	*Inferential statistics*	RR*
*Age: 45–64*	17.2%^a^	9.2%^b^	5.6%^b^	*X*^2^(2, 2,572) = 36.10, *p* < 0.001	3.07
*Age: 65+*	7.2%^a^	5.1%^a^	6.2%^a^	*X*^2^(2, 1,690) = 2.32, *p* = 0.314	1.16
**Education**					
	*Low education*	*Medium education*	*High education*	*Inferential statistics*	RR*
*Age: 45–64*	15.3%^a^	10.0%^b^	6.5%^c^	*X*^2^(2, 2,572) = 18.54, *p* < 0.001	2.35
*Age: 65+*	7.4%^a^	4.5%^a^	7.5%^a^	*X*^2^(2, 1,690) = 6.23, *p* = 0.044	0.99
**Job status**					
	*Low job status*	*Medium job status*	*High job status*	*Inferential statistics*	RR*
*Age: 45–64*	16.2%^a^	8.7%^b^	7.6%^b^	*X*^2^(2, 2,572) = 49.41, *p* < 0.001	2.20
*Age: 65+*	6.6%^a^	5.6%^a^	5.6%^a^	*X*^2^(2, 1,690) = 0.59, *p* = 0.742	1.18
**Income**					
	*Low income*	*Medium income*	*High income*	*Inferential statistics*	RR*
*Age: 45–64*	17.5%^a^	8.9%^b^	4.9%^c^	*X*^2^(2, 2,572) = 49.41, *p* < 0.001	3.57
*Age: 65+*	8.4%^a^	5.0%^b^	3.9%^a,b^	*X*^2^(2, 1,690) = 8.48, *p* = 0.014	2.15

Differing superscript letter per row denote significant differences at uncorrected *p* < 0.05 for frequency rates across respective low, middle, or high strata; the same superscript letter conversely denote non-significant differences. *RR, risk ratio for the comparison of categories with low vs. high SES; n_age group_ 45–64 = 2,572 and n_age group_ 65 + =1,690.

In middle-aged males, the total SES-Index, job status, and income, exhibited differential frequency rates of depression with descriptively decreasing rates across low, medium, and high expressions of the respective SES variable, whereby all SES dimensions revealed a strong ‘social gradient’ concerning depression frequency with RRs > 4.0 for low vs. high expressions of SES (Table 4). Education played a comparatively small role for depression prevalence in middle-aged males. In older males, no significant differences in depression frequency rates by SES-groups (total index, education, job status, income) were found, suggesting SES being less relevant for depression rates in older age.

**Table 4:** Depression frequency rates (%) by age and socio-economic status categories in males (*n* = 4,138).

Total SES-Index					
	*Low total SES*	*Medium SES*	*High SES*	*Inferential statistics*	RR*
*Age: 45–64*	9.9%^a^	6.0%^b^	2.1%^c^	*X*^2^(2, 2,289) = 26.58, *p* < 0.001	4.71
*Age: 65+*	3.1%^a^	2.6%^a^	1.5%^a^	*X*^2^(2, 1849) = 2.32, *p* = 0.314	2.07
**Education**					
	*Low education*	*Medium education*	*High education*	*Inferential statistics*	RR*
*Age: 45–64*	7.1%^a^	6.4%^a^	2.9%^b^	*X*^2^(2, 2,289) = 11.32, *p* = 0.003	2.45
*Age: 65+*	2.2%^a^	2.9%^a^	1.9%^a^	*X*^2^(2, 1849) = 1.30, *p* = 0.521	1.16
**Job status**					
	*Low job status*	*Medium job status*	*High job status*	*Inferential statistics*	RR*
*Age: 45–64*	10.0%^a^	5.4%^b^	2.4%^c^	*X*^2^(2, 2,289) = 21.92, *p* < 0.001	4.17
*Age: 65+*	3.1%^a^	2.8%^a^	1.4%^a^	*X*^2^(2, 1849) = 3.50, *p* = 0.174	2.21
**Income**					
	*Low income*	*Medium income*	*High income*	*Inferential statistics*	RR*
*Age: 45–64*	11.3%^a^	5.5%^b^	2.3%^c^	*X*^2^(2, 2,289) = 37.53, *p* < 0.001	4.91
*Age: 65+*	4.2%^a^	2.1%^a^	1.8%^a^	*X*^2^(2, 1849) = 5.78, *p* = 0.056	2.33

Differing superscript letter per row denote significant differences at uncorrected *p* < 0.05 for frequency rates across respective low, middle, or high strata; the same superscript letter conversely denote non-significant differences. *RR, risk ratio for the comparison of categories with low vs. high SES; n_age group 45–64_ = 2,289 and n_age group 65+_ = 1,849.

Descriptively, males seemed to exhibit stronger RRs related to low vs. high SES status groups in middle age (all RRs > 4) than females (all RRs between 2.35 to 3.57).

### Robustness and relevance of SES sub-scores including additional SES-variables and somatic symptoms

Detailed results of the additional analyses can be found in Supplementary Tables 3, 4. The regression model including age + sex all HCHS-SES sub-scores explained 5.7% of variance. Adding the SES variables marital status and housing type, which were both significant, lead to a slight increase in explained variance (R^2^_adj_ = 0.072) and an improvement in model fit based on BIC (∆BIC_2-1_ = −85; Supplementary Table 4), while job status (non-significant trend) and income remained significant. In comparison, model 3, which included somatic symptoms in addition to the new SES variables, had a substantially better fit (∆BIC_3-1_ = −3,814; ∆BIC_3-2_ = −3,729) and explained a substantially higher amount of variance (R^2^_adj_ = 0.485). Income remained significant, as well as housing and marital status, whereas job status was no longer significant, but now instead education became highly significant.

## Discussion

Overall, the present study suggests that SES is significantly associated with levels of depression in middle-to-old-age. However, the amount of explained variance by SES in addition to age and sex is rather low (0.4–2%). The best performing models in terms of goodness of fit and explained variance involved *job status* and *income,* or *income* alone as factor(s; with age and sex as covariates; 5.8–5.9% explained variance), hinting toward the central role of income for current levels of depression in the HCHS sample. The utility of the total SES-Index and its’ sub-dimensions (education, job/ occupational status, income) was demonstrated examining depression frequency rates across socioeconomic strata (low, medium, high), as well as sex and age groups (45–64 years vs. 65+ years). Depression frequency rates were significantly lower in older (4.1%) compared to middle- (7.9%) aged, and lower in male (4.2%) compared to female (8.4%) participants. In both middle-aged females and males, low (17.2 and 9.9%) vs. high (5.6 and 2.1%) total SES-Index (RRs = 3.07 and 4.71), as well was low (17.5 and 11.3%) vs. high (4.9 and 2.3%) income (RRs = 3.57 and 4.91) were related to differential depression frequency rates. Depression frequency rates in middle-aged women differed by levels of education, but this was not the case for men. In older adults, generally no differences in depression frequency rates across SES variables and strata were observed, except for income, whereby the effect was restricted to females (low income: 8.4%, high income: 3.9% depression frequency rates; RR = 2.15). Middle-aged males showed descriptively stronger discrepancies in depression risk related to pertaining to low vs. high SES groups as compared to middle-aged females.

First, the prominent role of (net equivalent household) income (index sub-score) across analyses in the present study seems noteworthy. Higher income was both the most relevant predictor in the regression analyses, and low-, medium-, and high-income classes most strongly discriminated depression frequency rates across both females and males, both in middle as well as (restricted to women) old age. Similarly, in a German sample (*n* = 12,484, age 35–74 years) household net-income was identified as an independent predictor of depressive symptoms at a 30 month follow up in those individuals without depressive symptoms at baseline (42). Even in a German study conducted from 2000 to 2001 only income was related to depression, whereas education, occupation, or homeownership where inconsistently related or unrelated (43). The replication and consistency of income as a relevant predictor for depression levels at different points in time with different German samples may suggest that income is particularly relevant for social inequality in mental health in Germany, but more research is needed to confirm this assumption. The results could speak for a materialistic explanation for differential depression frequency rates across social strata. Within this explanatory framework, income determines a person’s access to goods and services relevant for mental health and the degree of (potential protection from) exposure to environmental stressors. The materialistic view on mental health suggests the necessity to combat inequality at a structural level, such as by urban planning, social policies and welfare, thereby protecting poorer individuals from social and environmental hazards and improving access to relevant resources (44). Interestingly, in older age (65+), income was descriptively more relevant for females compared to males in terms of differential depression rates across income strata. This confirms research suggesting that poverty in old age strikes women harder (45).

Second, the finding that education was no significant predictor when added concurrently in a regression model with (age + sex +) job status and income, is noticeable. Education represents immaterial resources as, for example, knowledge and cognitive skills that relate to health literacy and health-related behavior (46). Educational attainment has been associated with health outcomes (46) and more specifically with mood disorders (47), and depressive symptoms (16, 20). However, studies which compared the relative association between different SES sub-dimensions with depressive symptoms have pointed toward a less prominent role of education. A British meta-analysis, which compared the prognostic performance of employment, financial distress, housing status, and education concerning depression, showed that education and financial stress were not associated with prognosis after the model had been adjusted for other prognostic factors (17). In a German longitudinal study (30 months) education only predicted reduced experiences of anhedonia and depressed mood in those individuals who did not report these symptoms at baseline (OR = 0.95, 95% CI[0.93, 0.97]) (47). The discrepancy in results regarding the role of education in depression may arise from differences in measures (16, 48). Studies that do differentiate between sub-dimensions of SES do not necessarily include the same or all three sub-dimensions of SES (48). In addition, education has been conceptualized for example by years of education (20), or highest level of education with differing numbers and content of levels (16, 17, 46, 47).

A critical issue overall is the small amount of explained variance by SES in the current paper (~5–7% across models that included age + sex + different SES variables). In an additional exploratory analysis (see SM), we tested whether the previously identified relevant SES sub-scores job status and income would remain significant upon inclusion of additional SES variables (marital status, housing type) and somatic symptoms, and how much variance would be explained by additional SES variables vs. somatic symptoms. It was discovered, that adding additional SES variables slightly improved model fit and explained variance, while including somatic symptoms lead to a substantial improvement in model fit and explained variance, with now 48.5% overall explained variance. Albeit causality cannot be inferred from any of the analyses, the additional analyses suggest that somatic symptoms and depressiveness are tightly related, whereas SES variables may explain a rather modest amount of variance.

### Limitations

Generally, SES and its association with (mental) health variables are likely to be influenced by cultural and historical factors. Hence, the representativeness of the current regional (Hamburg, Germany) sample for the association between SES, especially income, and depressive symptoms *per se*, is likely influenced by specific regional factors. Furthermore, the direction of the association remains unclear. Both social causation (i.e., socioeconomic disadvantages leading to worse mental health status) and social selection or decline (i.e., mental illness or related vulnerability leading to lower socioeconomic status) need to be discussed, whereby reciprocal associations are likely, albeit social causation has previously been discussed as having better empirical support (49). Recently, economic selection has been introduced as a third hypothesis, whereby it is stated that the trajectory of health and the economic condition of an individual are determined by the initial economic status. Thereby, if initial economic status is low, an accumulation of disadvantages in terms of ‘path dependence’ or a ‘locked life trajectory’ with ongoing socioeconomic deprivation and related health disparities can take place. A Chinese study on self-rated depression in adults aged 45 and above has shown that all three hypotheses are statistically supported using structural equation modeling (50). Another point of criticism is the fact that subjective SES was not assessed, which captures the awareness and social comparison dimension of one’s SES and which has been shown to independently contribute to (mental) health (51). This could have been achieved, e.g., by using the single-item social ladder (52), or assessing education-, occupation-, and income- ladders separately (53). Moreover, the SES-Index sub-score income had 33.9% missing values which is a high missingness rate for data imputation. However, individual income, which is closely related, had less than 25% missing data and proved to be a significant predictor in the model as well. In addition, in the present study, clinically relevant levels of depression were inferred from a cut-off based on the PHQ-9 self-report screening tool, rather than clinician ratings using a diagnostic tool to infer a true diagnosis. Nevertheless, using the same cut-off for the PHQ-9 (≥ 10), a representative German national health study (DEGS1) reported slightly higher depression rates (middle age [45–64]: 8.9%; older age [65–79]: 5.5%) (23) than the frequency rates found in the current sample (7.9 and 4.1%). Besides the fact that time of assessment differed between DEGS1 (2008–2011) and the present (2016–2018) sample, it is likely, that the HCHS sample is not representative for the German population. The city of Hamburg is particularly wealthy with, for example, the highest gross domestic product per person employed in 2021 (54), and the third highest household income in 2020 (55) among all German states. Lastly, albeit the focus of the present paper was to both establish and examine a standard SES-Index measure including its sub-dimensions and assess their explanatory potential concerning depressive symptoms, interactions are likely to occur between different socioeconomic dimensions, such as posing a potentiated burden for those affected by multiple socioeconomic disadvantages. Future studies should include more SES variables and study interactions between them.

## Conclusion

The results of the present study suggest that SES is significantly related to levels of depression in middle-to-old age – albeit SES only explains a small amount of variance, speaking for other factors being of more relevance. The standardized total SES-Index significantly explained variance over and above age and sex, whereby only the index sub-dimensions *income* and *job status* were significant predictors and *education* was not. Overall, income was the strongest and most robust SES sub-dimension in explaining depressive symptoms. Furthermore, both common and divergent patterns of depression frequency rates across different social-economic strata were identified for females vs. males, and middle-aged vs. older individuals. Older age was generally associated with lower, and female sex with higher depression frequency rates. Income discrepancies were the strongest determinants for differential frequency rates of depression, with relative risks for low-income individuals being three to five times higher than for high income in middle-aged individuals (45–64). In older age (65+), generally there were no significant differences in frequency rates across social-economic strata, except again for income, but restricted to females. Middle-aged males in general showed descriptively stronger relative risk related to low SES compared to females concerning depression frequency rates, suggesting that low social rank in general may deem males more vulnerable to depression than females.

For future studies within HCHS and beyond, the standardized approach to SES as presented in the present study is highly recommended, whereby the SES sub-dimensions as well as sex- and age-differences should be examined. Studies aiming at a more detailed examination of SES influencing specific outcomes of interest should consider adding subjective SES. Studies rather interested in only controlling for or evaluating SES on a global scale (covariate level) could use the total SES-Index.

## Data Availability

The data analyzed in this study are subject to the following licenses/restrictions: the data that support the findings of this study are available from the Hamburg City Health Study Center, but restrictions apply to the availability of these data, which were used under license for the current study, and so are not publicly available. Data are however available upon reasonable request and with permission of the Hamburg City Health Study Center. Requests to access these datasets should be directed to Leonie Ascone, l.ascone-michelis@uke.de.

## Footnotes

^1^https://github.com/AnneKlimesch/hchs_ses_index
