# Supplementary material for: The differential role of socioeconomic status dimensions in depressive symptoms of aging adults: data from the Hamburg City Health cohort Study
Source: Front Public Health. 2024 Aug 29;12:1430325. doi: 10.3389/fpubh.2024.1430325 (PMC11390532; doi:10.3389/fpubh.2024.1430325)
Supplement: Supplementary file 1 [file Data_Sheet_1.docx]

**Supplementary Material**

**Section 1. SES-Index validation**

A principal component analysis (PCA) was performed to determine whether the three index sub-scores (*education*, *job status*, *income*) loaded on a single factor. In addition, intercorrelations between the sub-scores in the original sample, imputed data, and final (combined) sample were compared as an additional validation of the SES-Index. The PCA (no rotation, method = correlation) resulted in an Eigenvalue greater than 1 for the first factor only. The scree plot (Figure 1) indicated that all three sub-scores are best explained by a single factor accounting for 64.8% of the variance. The three SES-Index sub-scores loaded on the factor with communalities between 0.742 and 0.841. Spearman rho intercorrelations (pairwise exclusion of missing values) between the index sub-scores were, as expected, moderate to large (education and job status [n= ] ρ = .599; education and income ρ = .467; job status and income ρ = .467; all p < .001). The factor solution and intercorrelations of the SES-Index sub-scores were replicated with the imputed data only (n = 3,976; education and job status r = .521; education and income r = .465 job status and income r = .564; all p < .001) as well as in the final sample (main text, Table 1).


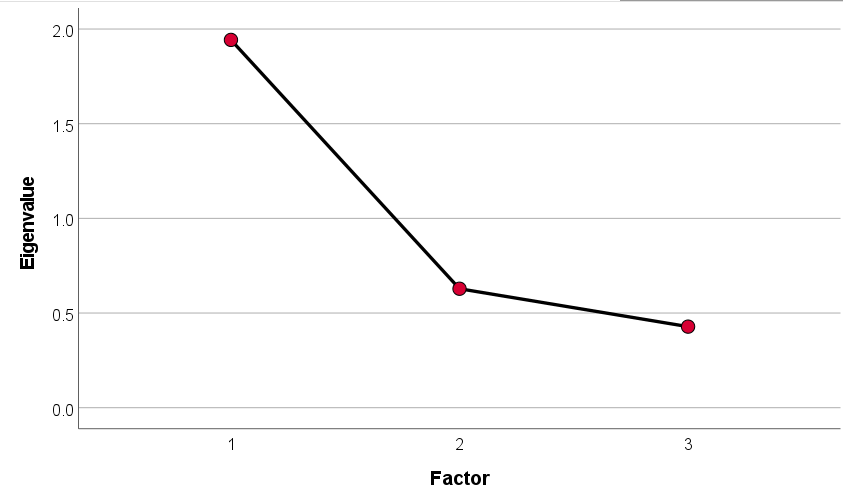


**Supplementary figure 1.** Principal component analysis scree plot with the SES-Index sub-scores *education*, *job status*, and *income.*

**Section 2. SES-Index sub-score formation**

| **Supplementary table 1.1.** School and vocational degrees sorted by ordinal code. | | | |
| --- | --- | --- | --- |
| **Ordinal**  **Code** | **Highest school degree**  *Which is your highest school degree?* | **Ordinal**  **Code** | **Highest professional degree**  *Which professional degrees have you acquired?* |
| **1a** | In school/ visiting a general school (full-time) | **1a** | None/ not involved in any kind of professional training |
| **1b** | Left school without a degree | **1b** | Currently in professional training (trainee, student, etc.) |
| **2a** | Lower school degree (9 years; ‘Hauptschule’) | **1c** | In technical school/training |
| **2b** | Poli-technical school degree of the German Democratic Republic (8/9 years) | **2** | Completed vocational/ practical training |
| **3a** | Middle school degree (10 years, ‘Realschule’) | **3** | Completed vocational school (e.g., commercial school preparation for civil service or administration) |
| **3b** | Poli-technical school of the German Democratic Republic (degree after 10 years) | **4a** | Finished technical (master) school in the German Democratic Republic |
| **4** | Technical college entry qualification | **4b** | Completed technical or other advanced vocational master training |
| **5a** | A-levels/ high-school diploma (12-13 years) | **5** | Bachelor’s degree at advanced (technical) colleges/ university |
| **5b** | A-levels/ high-school diploma (12-13 years; acquired through second chance education) | **6** | Master’s degree at advanced (technical) colleges |
| - | - | **7a** | Master’s degree or diploma |
| - | - | **7b** | PhD/ university doctoral degree |

| **Supplementary table 1.2.** Coding system for the SES-Index sub-score *education* sorted by index value. | | |
| --- | --- | --- |
| **Highest school degree** | **Highest professional degree** | **Index value**  (GEDA(1)-oriented) |
| 1a/ 1b | 1a / 1b/ 1c | 1.0 |
| 2a | 1a/ 1b/ 1c | 1.7 |
| 2b/ 3a/ 3b | 1a/ 1b/ 1c | 2.8 |
| 1b/ 2a | 2/ 3/ 4a/ 4b | 3.0 |
| 2b/ 3a/ 3b | 2/ 3/ 4a/ 4b | 3.6 |
| 4/ 5a/ 5b | 1a/ 1b/ 1c | 3.7 |
| 4/ 5a/ 5b | 2/ 3/ 4a/ 4b | 4.8 |
| 4/ 5a/ 5b | 5/ 6 | 6.1 |
| 4/ 5a/ 5b | 7a/ 7b | 7 |

| **Supplementary table 1.3.** Coding system for the SES-Index sub-score *job status* sorted by ordinal code. | | | | | | | |
| --- | --- | --- | --- | --- | --- | --- | --- |
| **Ordinal**  **Code** | **Occupational category**  *Which is your current job position (or, if currently not working, last position)?* | | **Occupational level/ rank**  *Specification of the level or rank of the occupation, corresponding to the selected occupational category* | | | | **Index Value** (DEGS1 (2)-oriented) |
| **1a** | Independent farmer or farmer in a cooperative society | | Agricultural area < 10ha | | | | 1.1 |
|  |  |  | Agricultural area ≥ 10ha | | | | 1.0 |
|  |  |  | Member of cooperative society | | | |  |
| **1b** | Currently in professional training (trainee, student, etc.) | | In business and administrative tasks | | | | 1.0 |
|  |  |  | In trades/ commercial tasks | | | |  |
|  |  |  | Other | | | |  |
| **2a** | Worker | | Unlearned worker | | | | 1.3 |
|  |  |  | Foreman/ - woman/ leading worker | | | | 2.0 |
|  |  |  | Learned worker | | | | 2.1 |
|  |  |  | Skilled/ expertise worker | | | |  |
|  |  |  | Master craftsman/-woman, overseer, team leader | | | | 2.4 |
| **2b** | Helping family member | | Not further specified | | | | 2.4 |
| **3a** | Independent/ self-employed/ (small) business owner in the craft sector, trade, industry | | No employees | | | | 3.5 |
|  |  |  | 1-4 employees | | | | 3.6 |
|  |  |  | 5+ employees | | | | 4.2 |
|  |  |  | Member of a personal trading company | | | |  |
| **3b** | Contract employee, staffer | | Executive tasks following general instructions | | | | 2.4 |
|  |  |  | Qualified tasks following general instructions | | | | 3.6 |
|  |  |  | Independent, self-responsible tasks or with task-related responsibility for other personnel | | | | 4.2 |
|  |  |  | Extensive management tasks with decision authority | | | | 4.7 |
| **4** | Official/ civil servant, judge, military official | | Simple/ lower public service | | | | 2.9 |
|  |  |  | Middle public service | | | | 4.1 |
|  |  |  | Higher public service | | | | 5.2 |
|  |  |  | Highest public service | | | | 6.4 |
| **5** | Academic of the liberal professions (e.g., medical doctor, lawyer, tax consultant, etc.) | | No further employees | | | | 6.2 |
|  |  |  | 1-4 employees | | | | 6.8 |
|  |  |  | 5+ employees | | | | 7 |
| **Supplementary table 1.4.** Coding system for the SES-Index sub-score *income.* | | | | | |  |  |
| **Net equivalent income range** | | **N** | | **%** | **Index**  **value** |  |  |
| ≤ 1099.99 | | 505 | | 7.6 | 1 |  |  |
| 1100.00 – 1374.99 | | 397 | | 6.0 | 1.5 |  |  |
| 1375.00 – 1583.332 | | 462 | | 7.0 | 2 |  |  |
| 1583.333 – 1833.332 | | 574 | | 8.7 | 2.5 |  |  |
| 1833.333 – 1874.99 | | 428 | | 6.5 | 3 |  |  |
| 1875.00 – 2166.66 | | 603 | | 9.1 | 3.5 |  |  |
| 2166.67 – 2374.99 | | 544 | | 8.2 | 4 |  |  |
| 2375.00 – 2500.00 | | 598 | | 9.1 | 4.5 |  |  |
| 2500.01 – 2833.32 | | 318 | | 4.8 | 5 |  |  |
| 2833.33 – 3166.66 | | 414 | | 6.3 | 5.5 |  |  |
| 3166.67 – 3666.66 | | 597 | | 9.0 | 6 |  |  |
| 3666.67 – 4666.66 | | 561 | | 8.5 | 6.5 |  |  |
| ≥ 4666.67 | | 605 | | 9.2 | 7 |  |  |
| -999 | | 3,389 | | 33.9 | -999 |  |  |

**Section 3. Sample characteristics**

| **Supplementary table 2.1.** Pattern of missing data for variables assessing SES. | | | | | | | | | | | |
| --- | --- | --- | --- | --- | --- | --- | --- | --- | --- | --- | --- |
| **Missing value category** | | **N missing values** | | | | | | | | | |
|  | | Sub-score education | | Sub-score job status | | Sub-score income | | | | | |
|  | | School education | Professional education | Occupational category | Occupational level/ rank | Household net income | | | Household size | No. adults including self | |
| do not wish to reply | | 94 | 168 | 111 | - | 1,819 | | | 90 | 133 | |
| do not know | | 16 | 33 | 112 | - | 186 | | | 4 | 16 | |
| n/a | | 628 | 592 | 999 | - | 725 | | | 510 | 813 | |
| -99 | | 29 | 41 | 103 | - | 47 | | | 20 | 87 | |
| n/p | | - | - | - | - | - | | | 84 | 355 | |
| other school degree/ other professional qualification | | 60 | 65 | - | - | - | | | - | - | |
| **Missing/**  **Not possible to compute subscore** | | **827** | **899** | **2,367** | **1,042** | **2,777** | | | **708** | **1,404** | |
|  |  | **1,412** | | **2,367** | | **3,394** | | | | | |
| **Note.** The table refers to the original sample (n = 10,000). n/a and -99 were coded by the HCHS study center, when questions were not filled out. Originally, n = 157 participants indicated they had *‘[an]other school degree’* and n = 320 indicated that they had *‘[an]other professional qualification’* but n=97 and n = 255 of those respectively were assigned to a valid category based on free text entries of the participant. | | | | | | | | | | | |
| **Supplementary table 2.2.** Pattern of missing data for occupational category and level/ rank specifications. | | | | | | | | | | |  |
| **Ordinal**  **Code** | **Occupational category**  (current or last) | | | | | | **n** | **n missing (n existing)**  occupational level/ rank specification | | |  |
| **1a** | Independent farmer or farmer in a cooperative society | | | | | | 46 | 40 (6) | | |  |
| **1b** | Currently in professional training (trainee, student, etc.) | | | | | | 1 | 1 (0) | | |  |
| **2a** | Worker | | | | | | 649 | 68 (581) | | |  |
| **2b** | Helping family member | | | | | | 41 | 0 (41) | | |  |
| **3a** | Independent/ self-employed/ (small) business owner in the craft sector, trade, industry | | | | | | 956 | 94 (862) | | |  |
| **3b** | Contract employee, staffer | | | | | | 5,645 | 720 (4,925) | | |  |
| **4** | Official/ civil servant, judge, military official | | | | | | 834 | 69 (765) | | |  |
| **5** | Academic of the liberal professions (e.g., medical doctor, lawyer, tax consultant, etc.) | | | | | | 503 | 50 (453) | | |  |
| **-999** | Missing | | | | | | 1,325 | **-** | | |  |
| **sum** | **-** | | | | | | **10,000** | **1,042 (7,633)** | | |  |

| **Supplementary table 2.3.** Cut-off values of SES-Index categories (n = 8,400). | | | | | |
| --- | --- | --- | --- | --- | --- |
| **Variable** | **Category** | **Quintile** | **Lower bound** | **Upper bound** | **%** |
| SES-Index | Low | 1^st^ quintile | 3.00 | 9.649 | 20.0 |
|  | Medium | 2^nd^ quintile | 9.65 | 11.29 | 20.0 |
|  |  | 3^rd^ quintile | 11.30 | 13.19 | 18.8 |
|  |  | 4^th^ quintile | 13.20 | 16.1839 | 21.3 |
|  | High | 5^th^ quintile | 16.1840 | 21.00 | 20.0 |
| Sub-score education | Low | 1^st^ quintile | 1.00 | 3.00 | 21.5 |
|  | Medium | 2^nd^ quintile | 3.01 | 3.60 | 24.8 |
|  |  | 3^rd^ quintile | 3.61 | 4.80 | 18.5 |
|  |  | 4^th^ quintile | 4.81 | 6.99 | 13.3 |
|  | High | 5^th^ quintile | 7.00 | 7.00 | 21.9 |
| Sub-score job status | Low | 1^st^ quintile | 1.00 | 3.49 | 18.8 |
|  | Medium | 2^nd^ quintile | 3.50 | 3.60 | 30.4 |
|  |  | 3^rd^ quintile | 3.61 | 4.19 | 8.5 |
|  |  | 4^th^ quintile | 4.20 | 4.20 | 24.2 |
|  | High | 5^th^ quintile | 4.21 | 7.00 | 18.2 |
| Sub-score income | Low | 1^st^ quintile | 1.00 | 2.50 | 21.1 |
|  | Medium | 2^nd^ quintile | 2.51 | 3.49 | 12.4 |
|  |  | 3^rd^ quintile | 3.50 | 4.50 | 31.3 |
|  |  | 4^th^ quintile | 4.51 | 5.99 | 15.0 |
|  | High | 5^th^ quintile | 6.00 | 7.00 | 20.1 |

**Additional exploratory analyses (3): Robustness and relevance of SES sub-scores including additional SES-variables and somatic symptoms.**

Additional analyses were run to (a) check whether there were potentially more SES variables relevant in explaining variance in depressive symptoms and (b) to put the amount of explained variance by the SES sub-dimensions and other SES variables into perspective (i.e., *relative relevance* of the SES sub-dimensions vs. an established, strongly associated risk-factor for depression) as well as to check whether SES remained to be significant (*robustness* of SES in explaining variance in depressive symptoms), after including somatic symptoms.

We excluded all cases that had missing values on these variables and merged them with the SES data of the main analysis which led to a sample of n = 6,341 (n(male) = 2,758; n(female) = 3,583, age = 46-78, PHQ-9 sample mean and standard deviation (M = 5.52, SD = 3.44)). Checking the assumptions for linear regression revealed no meaningful deviations or violations. However, the plots indicated heteroscedasticity which could narrow generalizability of the results to the population. Therefore, heteroscedasticity-consistent standard error estimators were applied. Before running the regression models, intercorrelations between all variables of interest were determined using non-parametric Spearman rho (ρ) correlations (see SM Table 3). All independent variables showed weak associations (ρ ≥ .10) with the dependent variable, except education (see SM Table 3 for details). Somatic symptoms, in line with our hypothesis, showed a strong correlation with depressive symptoms (ρ = .676).

The regression analyses (for details see SM Table 4) showed that including additional SES variables (housing, marital status; model 2) both improved model fit (BIC; compared to the simpler model that only included the SES dimensions education, job status, and income) and a small increase in explained variance was observed. Including somatic symptoms (model 3) however lead to a substantial improvement in both model fit and explained variance.

| **Supplementary table 3.** Intercorrelations (Spearman ρ) between all variables of interest (n = 6,341) | | | | | | | | |
| --- | --- | --- | --- | --- | --- | --- | --- | --- |
|  | **1.** | **2.** | **3.** | **4.** | **5.** | **6.** | **7.** | **8.** |
| 1. Age | - |  |  |  |  |  |  |  |
| 2. sex^1^ | -.025^*^ | - |  |  |  |  |  |  |
| 3. education | -.172^***^ | -.121^***^ | - |  |  |  |  |  |
| 4. job status | .039^**^ | -.170^***^ | .523^***^ | - |  |  |  |  |
| 5. income | -.095^***^ | -.164^***^ | .431^***^ | .465^***^ | - |  |  |  |
| 6. housing^2^ | .070^***^ | -.023 | .174^***^ | .178^***^ | .221^***^ | - |  |  |
| 7. marital status^3^ | .050^***^ | -.138^***^ | .049^***^ | .079^***^ | .155^***^ | .310^***^ | - |  |
| 8. som. sympt. (PHQ-15)^4^ | -.014 | .209^***^ | -.107^***^ | -.123^***^ | -.144^***^ | -.119^***^ | -.086^***^ | - |
| 9. depr. sympt. (PHQ-9)^5^ | -.134^***^ | .171^***^ | -.041^**^ | -.098^***^ | -.118^***^ | -.126^***^ | -.143^***^ | .676^***^ |
| **Note.** ^1^0 = male, 1 = female. ^2^marital status was assessed as 0=widowed/ single/ divorced, 1=married but not living together, 2=married and living together. ^3^housing was assessed as (0=renting, 1=property). ^4^som. sympt. = somatic symptoms, PHQ-15 = Patient Health Questionnaire-15 sum score. ^5^ depr. sympt. = depressive symptoms, PHQ-9 = Patient Health Questionnaire-9 sum score. * p < .05, ** p <.01, *** p < .001. | | | | | | | | |

| **Supplementary table 4.** Model summaries of additional multiple linear regression analyses  (n = 6,341). | | | | | | | | |
| --- | --- | --- | --- | --- | --- | --- | --- | --- |
| **Model summary** | ***B*** | ***95%CI[B]*** | ***robust***  ***S.E.*** | ***β*** | ***t*** | ***p*** | **R^2^_adj_** | ***BIC*** |
| **1** [F(5, 6335) = 78.19, p<.001] | | | | | | | .057 | 15351 |
| constant | 7.85 | [7.11 8.59] | .389 |  | 20.83 | <.001 |  |  |
| age | -.057 | [-.067 -.047] | .005 | -.137 | -10.89 | <.001 |  |  |
| sex^1^ | .876 | [.708 1.05] | .085 | .126 | 10.17 | <.001 |  |  |
| education | .016 | [-.051 -.082] | .035 | .007 | .457 | .684 |  |  |
| job status | -.082 | [-.180 .016] | .048 | -.025 | -1.64 | .100 |  |  |
| income | -.269 | [-.326 -.211] | .031 | -.129 | -9.18 | <.001 |  |  |
| **2** [F(8, 6332) = 71.85, p<.000] | | | | | | | .072 | 15266 |
| constant | 8.44 | [7.70 9.18] | .393 |  |  |  |  |  |
| Age | -.052 | [-.062 -.042] | .005 | -.125 | -9.96 | <.001 |  |  |
| sex | .828 | [.659 .997] | .085 | .119 | 9.61 | <.001 |  |  |
| education | .029 | [-.038 .095] | .035 | .013 | .843 | .399 |  |  |
| job status | -.068 | [-.165 .030] | .048 | -.021 | -1.36 | .173 |  |  |
| income | -.215 | [-.273 -.157] | .031 | -.103 | -7.28 | <.001 |  |  |
| housing^2^ | -.540 | [-.718 -.363] | .089 | -.078 | -5.97 | <.001 |  |  |
| marital status^3^ | -.290 | [-.383 -.197] | .048 | -.079 | -6.12 | <.001 |  |  |
| **3** [F(8, 6332) = 748.45, p<.000] | | | | | | | .485 | 11537 |
| constant | 4.06 | [3.49 4.63] | .289 |  | 14.05 | <.001 |  |  |
| age | -.048 | [-.055 -.040] | .004 | -.115 | -12.32 | <.001 |  |  |
| sex^1^ | .019 | [-.109 .146] | .065 | .003 | .284 | .777 |  |  |
| education | .084 | [.035 .133] | .026 | .038 | 3.33 | <.001 |  |  |
| job status | .005 | [-.068 .077] | .037 | -.001 | .128 | .898 |  |  |
| income | -.109 | [-.153 -.066] | .022 | -.053 | -4.96 | <.001 |  |  |
| housing | -.136 | [-.269 -.004] | .067 | -.020 | -2.02 | .044 |  |  |
| marital status | -.248 | [-.317 -.179] | .036 | -.068 | -7.03 | <.001 |  |  |
| som. sympt.^4^ | .605 | [.588 .621] | .011 | .666 | 71.32 | <.001 |  |  |
| **Note.** The dependent variable of both models was PHQ-9 score. All models were fitted based on the same sample (n = 6,341). The *t*-statistics, *p*-values, and *CIs* were estimated based on robust standard errors. BIC = Bayesian Information Criterion. ^1^0 = male, 1 = female. ^2^ marital status was assessed as 0 = widowed/ single/ divorced, 1=married but not living together, 2 = married and living together. ^3^ housing was assessed as (0 = renting, 1 = property). ^4^som. sympt. = somatic symptoms, assessed with the Patient Health Questionnaire 15 (PHQ15). | | | | | | | | |

**References**

1. Lampert T, Kroll LE, Müters S, Stolzenberg H. *Sozioökonomischer Status und Gesundheit*. [Socioeconomic status and health]. Bundesgesundheitsblatt. 2013;56:814–821. h

2. Lampert T, Kroll L, Müters S, Stolzenberg H. *Messung des sozioökonomischen Status in der Studie zur Gesundheit Erwachsener in Deutschland (DEGS1)*. [Measurement of socioeconomic status in the German health interview and examination survey for adults (DEGS1)]. Bundesgesundheitsblatt - Gesundheitsforschung - Gesundheitsschutz. 2013;56:631-636. https://doi.org.10.1007/s00103-012-1663-4
